# Volatile anesthetics affect macrophage phagocytosis

**DOI:** 10.1371/journal.pone.0216163

**Published:** 2019-05-09

**Authors:** Hui Zha, Erika Matsunami, Nathan Blazon-Brown, Sophia Koutsogiannaki, Lifei Hou, Weiming Bu, Hasan Babazada, Kirsten C. Odegard, Renyu Liu, Roderic G. Eckenhoff, Koichi Yuki

**Affiliations:** 1 Department of Anesthesiology, Critical Care and Pain Medicine, Cardiac Anesthesia Division, Boston Children’s Hospital, Boston, Massachusetts, United States of America; 2 Department of Anaesthesia, Harvard Medical School, Boston, Massachusetts, United States of America; 3 Department of Pediatrics, Union Hospital, Tonji Medical College, Huazhong University of Science and Technology, Wuhan, China; 4 Department of Anesthesia, Kawasaki Saiwai Hospital, Kawasaki, Kanagawa, Japan; 5 Department of Anesthesiology and Critical Care, University of Pennsylvania, Philadelphia, Pennsylvania, United States of America; Massachusetts General Hospital, UNITED STATES

## Abstract

**Background:**

Perioperative infections, particularly surgical site infections pose significant morbidity and mortality. Phagocytosis is a critical step for microbial eradication. We examined the effect of commonly used anesthetics on macrophage phagocytosis and its mechanism.

**Methods:**

The effect of anesthetics (isoflurane, sevoflurane, propofol) on macrophage phagocytosis was tested using RAW264.7 mouse cells, mouse peritoneal macrophages, and THP-1 human cells. Either opsonized sheep erythrocytes or fluorescent labeled *Escherichia coli* were used as phagocytic objects. The activation of Rap1, a critical protein in phagocytosis was assessed using the active Rap1 pull-down and detection kit. To examine anesthetic binding site(s) on Rap1, photolabeling experiments were performed using azi-isoflurane and azi-sevoflurane. The alanine scanning mutagenesis of Rap1 was performed to assess the role of anesthetic binding site in Rap1 activation and phagocytosis.

**Results:**

Macrophage phagocytosis was significantly attenuated by the exposure of isoflurane (50% reduction by 1% isoflurane) and sevoflurane (50% reduction by 1.5% sevoflurane), but not by propofol. Photolabeling experiments showed that sevoflurane directly bound to Rap1. Mutagenesis analysis demonstrated that the sevoflurane binding site affected Rap1 activation and macrophage phagocytosis.

**Conclusions:**

We showed that isoflurane and sevoflurane attenuated macrophage phagocytosis, but propofol did not. Our study showed for the first time that sevoflurane served as a novel small GTPase Rap1 inhibitor. The finding will further enrich our understanding of yet-to-be determined mechanism of volatile anesthetics and their off-target effects. The sevoflurane binding site was located outside the known Rap1 functional sites, indicating the discovery of a new functional site on Rap1 and this site would serve as a pocket for the development of novel Rap1 inhibitors.

## Introduction

Emerging evidences suggest that surgical anesthetics possess immunomodulatory effects [1–3]. General anesthesia is mainly provided by intravenous and/or volatile anesthetics. Some patients require postoperative management in the intensive care unit (ICU) and may continue to receive sedation and pain medications. The majority of sedatives in the ICU are intravenous medications. Lately volatile anesthetics are attracting attention as ICU sedatives due to their potentially favorable profiles for pulmonary gas exchange [4]. Perioperative infections, such as surgical site infections (SSIs), can result in significant morbidity, mortality, and financial burdens [5–8]. Therefore, it is extremely important to understand if anesthetics potentially affect the function of professional phagocytes.

**Fig 1:**
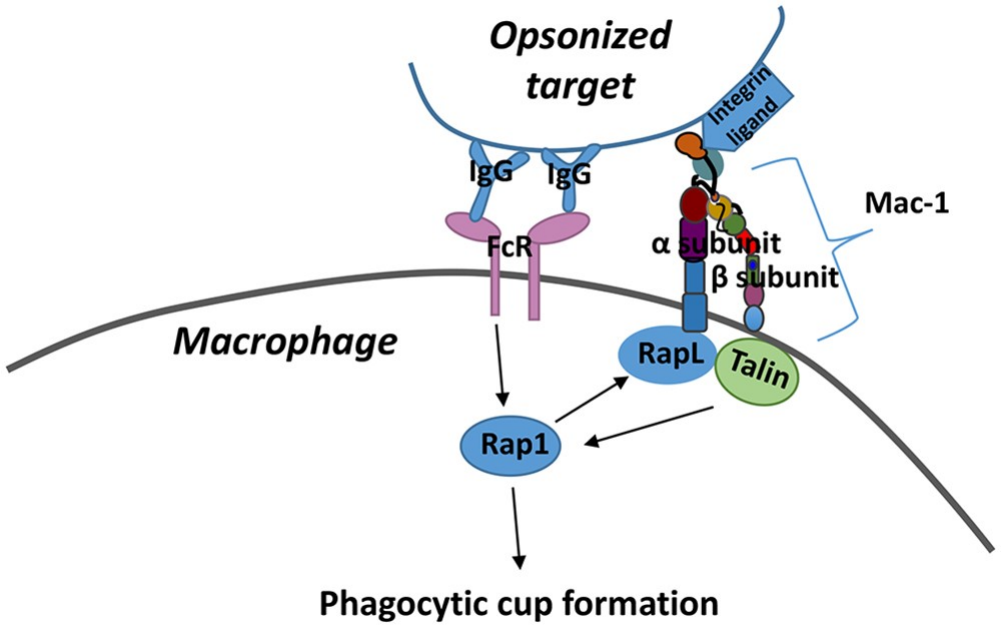
The effect of anesthetics on cross-talk of Fc receptor and Mac-1 for phagocytosis. Targets opsonized with IgG and Mac-1 ligand will be captured by macrophages via their Fc receptors (FcRs) and Mac-1. Mac-1 consists of α- and β-subunits as shown in the scheme. Rap 1 is activated by FcRs after FcRs engage with IgG. Activated Rap1 can activate protein called RapL, which interacts with α subunit of Mac-1. Activated Mac-1 can activate Rap1 via protein Talin. As shown in the scheme, Rap1 will receive signal from both FcRs and Mac-1 to form phagocytic cup.

Macrophages are professional phagocytes to efficiently remove invading microbes mainly by immunoglobulin G (IgG)- and complement-mediated phagocytosis [9, 10]. In complement-mediated phagocytosis, microbes opsonized non-specifically by complements bind to complement receptors (CRs) such as macrophage-1 antigen (Mac-1) and are ingested by macrophages with the help of additional signals. In IgG-mediated phagocytosis, microbes bound to specific IgG are ingested via constitutively active Fc receptors (FcRs). FcRs and complement receptors do not necessarily work independently, and usually cross-talk each other to augment overall phagocytosis process [11]. Microbes opsonized with IgG and Mac-1 ligand will be captured via FcRs and Mac-1. A Ras-like small GTPase Rap 1 is activated by signals from both FcRs and Mac-1 to form phagocytic cup, which is an invagination of the cell membrane that subsequently closes to form phagosomes (Fig 1). However, the effect of anesthetics on phagocytosis has not been examined with specific consideration to this cross-talk.

We aimed to study the role of different anesthetics (volatile anesthetics; isoflurane and sevoflurane, and intravenous anesthetic; propofol) in phagocytosis mediated by both FcR and CRs on macrophages [12]. In addition, we studied the underlying mechanism of the observed phenotype.

## Methods

### Cell culture and mice

RAW264.7 cells, THP-1 cells and mouse peritoneal macrophages were cultured in RPMI1640/ 10% fetal bovine serum (FBS), glutamate at 37°C, 5% CO_2_. Mice were purchased from Jackson Laboratory to obtain peritoneal macrophages (Bar Harbor, ME, USA). The protocol was approved by Boston Children’s Hospital IACUC, and the experimental procedure complies with institutional and federal guidelines regarding the use of animals in research. After mice were euthanized with CO_2_, peritoneal lavage was performed to collect cells. The cells were coated on culture dishes and subjected to washing in two hours. Remaining adherent cells were considered peritoneal macrophages. HEK293T cells were cultured in DMEM/10 mM HEPES/ 10% FBS, glutamate at 37°C, 5% CO_2_. RAW264.7, THP-1 and HEK293T cells were obtained from ATCC (Manassa, VA, USA).

### Sheep erythrocyte phagocytosis assay

RAW264.7 cells were seeded on 96-well flat culture plates at 5 x 10^5^ cells per well overnight. After washing, each well was given 100 μL of Na medium (5.6 mM glucose, 127 mM NaCl, 10.8 mM KCl, 2.4 mM KH_2_PO_4_, 1.6 mM MgSO_4_, 10 mM HEPES, 1.8 mM CaCl_2_; pH 7.3) [13]. Sheep erythrocytes (‘E’) (Colorado serum company; Denver, Colorado, USA) were opsonized with anti-sheep erythrocyte stroma antibody (Sigma-Aldrich; St. Louis, MO, USA) (‘E-Ig’) in Buffer A (HBSS, 15 mM HEPES, 1 mM MgCl_2_, 1 mM CaCl_2_; pH 7.3) for 30 minutes at room temperature. After washing, E-Ig were added to wells at the ratio of RAW264.7 cells: erythrocytes at 1:20. Cells were incubated with or without isoflurane (1%, 2%), sevoflurane (1.5%, 3%) or propofol (20, 100 μM) at 37°C for 30 minutes. For volatile anesthetic exposure, we used an air-tight chamber equilibrated with isoflurane or sevoflurane at different concentrations administered through a vaporizer (Fluotec; Cyprane Ltd., Keighley, UK). The chamber containing cells was kept in an incubator at 37°C. The concentration of volatile anesthetics was measured using an infrared spectroscopy (Ultima; Datex Instrument Corp., Helsinki, Finland). The concentrations tested here for both isoflurane and sevoflurane are clinically relevant, while the concentrations used for propofol are significantly well above clinical ranges. Propofol stock was dissolved in dimethyl sulfoxide (DMSO) solution, and the final DMSO concentration in samples exposed to propofol was 0.1%. Following incubation, erythrocytes not ingested by RAW264.7 cells were removed by washing and lysis. Remaining RAW264.7 cells were fixed with PBS/ 2% paraformaldehyde (PFA) and subjected to microscopy study. The number of erythrocytes was counted per 100 macrophages and reported as phagocytosis index. % phagocytosis was calculated as [(phagocytosis index of E-Ig without anesthetics)- (phagocytosis index of E-Ig with anesthetics)]/[(phagocytosis index of E-Ig)-(phagocytosis index of E)] x 100(%).

### Flow cytometry analysis of phagocytic receptor expression on RAW 264.7 cells

RAW264.7 cells were co-incubated with IgG-opsonized sheep erythrocytes. Following blocking with FcR antibody (anti-mouse CD16/CD32 antibody, clone 93; Biolegend, Dedham, MA, USA), CR Mac-1, CR4 (CD11c/CD18) and FcR FcγI on RAW264.7 cells were probed using phycoerythrin (PE) labeled anti-mouse CD11b antibody (clone M1/17), anti-mouse CD11c antibody (clone N418) and anti-mouse CD64 antibody (clone X54-5/7.1), respectively (all from Biolegend). FcR FcγII/III was detected with PE-labeled anti-mouse CD16/32 antibody (clone 93). Then cells were subjected to flow cytometry analysis. Sheep erythrocytes were gated out by forward scatter and negative CD11b expression.

### *Escherichia coli* (*E. coli*) phagocytosis assay

The phagocytosis of *E*. *coli* was examined using Phagotest kit (Glycotope Biotechnology; Berlin, Germany) per the company protocol with minor modification. Briefly, RAW264.7 cells, peritoneal macrophages, and THP-1 cells were adjusted to 5 x 10^6^ cells/mL in RPMI1640/10% FBS, and aliquoted with 5 x 10^5^ cells per sample. THP-1 cells were stimulated with phorbol 12-myristate 13-acetate (PMA, 100 ng/mL) for three days and then subjected to phagocytosis experiments. All the samples were incubated on ice for 10 min and given the pre-chilled *E*. *coli*-fluorescein (FITC) 20 μL (4 x 10^7^ *E*. *coli* -FITC). Samples were mixed and incubated for 30 min at 37°C. They were transferred on ice, quenched, and washed. Cells were suspended in PBS/1% PFA and subjected to flow cytometry analysis.

### Rap1 activation assay

Rap1 activation was measured using active Rap1 pull-down and detection kit (Thermo Fischer Scientific; Waltham, MA, USA). Briefly, RAW264.7 or HEK293T cells were lysed on ice and incubated with glutathione agarose beads coupled to GST-RalGDS (Ral guanine dissociation stimulator) to detect activated Rap1 [14]. After washing beads, samples were resolved by SDS- polyacrylamide gel and transferred to nitrocellulose membranes. After blocking in 3% bovine serum albumin (BSA), the membranes were probed with anti-Rap1 antibody (Thermo Fischer Scientific). Then, the membranes were incubated with anti-rabbit IgG horseradish peroxidase (HRP) conjugate (Thermo Fischer Scientific). Total Rap1 levels were determined using cell lysates that were not subjected to co-incubation with beads. Immunoreactive proteins were visualized by enhanced chemiluminescence and intensities were quantified with Image J software (National Institutes of Health; Bethesda, MD, USA).

### Protein purification

pGEX2T-Rap 1 plasmid was a gift from Dr. Julian Downward (London, UK; Addgene plasmid #55664) [15]. Rap1 protein was induced with 0.3 mM of isopropylthio-β-galactoside (ITPG) at 25 °C for 16 hours. Pellets were sonicated and supernatants were collected. Protein was purified using glutathione sepharose 4B beads (GE Healthcare; Chicago, IL, USA).

### Rap1 photolabeling experiments

Photolabeling of Rap1 protein using azi-isoflurane and azi-sevoflurane was performed as previously described [16]. Azi-isoflurane and azi-sevoflurane are photoaffinity probes, developed by incorporating a diazrinyl moiety (CHN_2_, 40 Da) into isoflurane and sevoflurane, respectively [17]. Azi-isoflurane or azi-sevoflurane (final concentration, 100 μM) was equilibrated with Rap1 protein (1 mg/mL) and 500 μM isoflurane or sevoflurane in a reaction volume of 300 μL for 10 min and then exposed to 300-nm light under a Rayonet RPR-3000 Lamp (Southern New England Ultraviolet Co.; Branford, CT, USA) in a 1-mm path-length quartz cuvette for 25 min. The protein was separated on an SDS-polyacrylamide gel and stained with Coomassie G-250. The protein gel band was excised for liquid chromatography (LC)-mass spectrometry (MS)/MS. After trypsin digestion, samples were injected into a nano-LC column with online electrospray into a LTQ linear ion trap (Thermo Fisher Scientific). Raw data were acquired with XCalibur (Thermo Fischer Scientific), and Sequest software (Scripps Research Institute, La Jolla, CA, USA) was used to search for b and y ions against the sequence of Rap1 for adducts of the appropriate mass (196 Da).

### Rigid docking analysis

Using the previously reported Rap1 structure (Protein Data Bank ID: 4KVG [18]), we performed the *in silico* docking of sevoflurane using Autodock Vina software (The Scripps Research Institute). The adducted residue by photolabeling experiment was chosen as a grid center. Top ranked docked site of sevoflurane was used.

### Mutagenesis and transient transfection of Rap1 to HEK 293 T cells

Rap1 wild type (WT) pcDNA3.1 plasmid was kindly given by Dr. Timothy Springer (Boston Children’s Hospital, Boston, MA, USA). Alanine scanning mutagenesis was performed using Quikchange XL kit (Agilent), and DNA sequences were confirmed. Plasmids were transfected into HEK 293T cells using Lipofectamine 2000 (Invitrogen, Carlsbad, CA, USA) according to the manufacture’s protocol.

### RAW264.7 cells stably transfected with Rap1 WT and mutant

After transfecting RAW264.7 cells with Rap1 WT and L161A mutant using Lipofectamine 2000, they were selected in RPMI1640/10% FBS, glutamate containing G418 (600 μg/mL) to obtain stable cell lines.

### Statistical analysis

Statistical analyses used were shown in individual figure legends. Statistical significance was defined as P< 0.05. All statistical calculations were performed using Prism 5 software (GraphPad Software, La Jolla, CA, USA).

## Results

### Isoflurane and sevoflurane attenuated macrophage phagocytosis, but propofol did not

**Fig 2:**
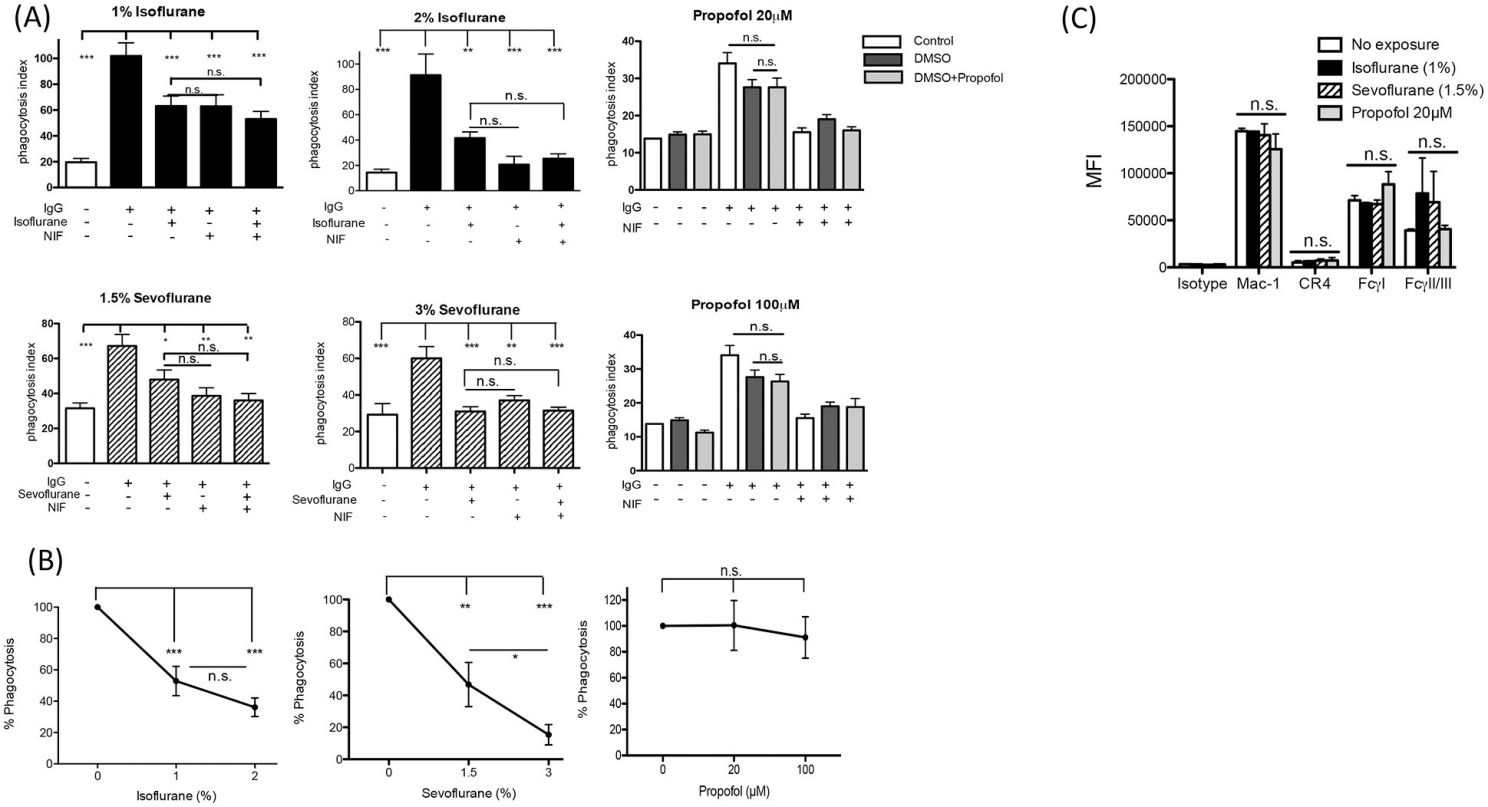
The effect of anesthetics on macrophage phagocytosis and receptor expression level. (A) Macrophage cell line RAW264.7 cells were used to test phagocytosis of sheep erythrocytes opsonized by IgG under different anesthetics (Isoflurane 1 and 2%, Sevoflurane 1.5 and 3%, Propofol 20 and 100 μM). Propofol was dissolved in DMSO. DMSO at the final concentration was 0.1%. Neutrophil inhibitory factor (NIF) is a Mac-1 inhibitor and used at 10 μM. Data are shown as mean +/- S.D. of quadruplicates. Representative figures from two independent experiments are shown. Statistical analysis was performed using one-way ANOVA with Bonferroni *post hoc* analysis. *, ** and *** represent p<0.05, p<0.01, and p<0.001, respectively. n.s. = not significant. (Β) Macrophage cell line RAW264.7 cells were used to test phagocytosis of sheep erythrocytes opsonized by IgG under different anesthetics. %Phagocytosis was calculated as in the Methods. Data are shown as mean +/- S.D. of quadruplicates. Representative figures from two independent experiments are shown. Statistical analysis was performed using one-way ANOVA with Bonferroni *post hoc* analysis. *, ** and *** represent p<0.05, p<0.01, and p<0.001, respectively. n.s. = not significant. (C) The expression levels of complement receptors and Fc receptors on RAW264.7 cells were detected by flow cytometry when co-incubated with IgG opsonized sheep erythrocytes as described in the method. Expression levels were analyzed with FlowJo software. Data are shown as mean +/- S.D. of quadruplicates. Representative figures from two independent experiments are shown. Statistical analysis was performed using one-way ANOVA with Bonferroni *post hoc* analysis. n.s. = not significant. CR4; complement receptor 4. MFI; mean fluorecense intensity.

**Fig 3:**
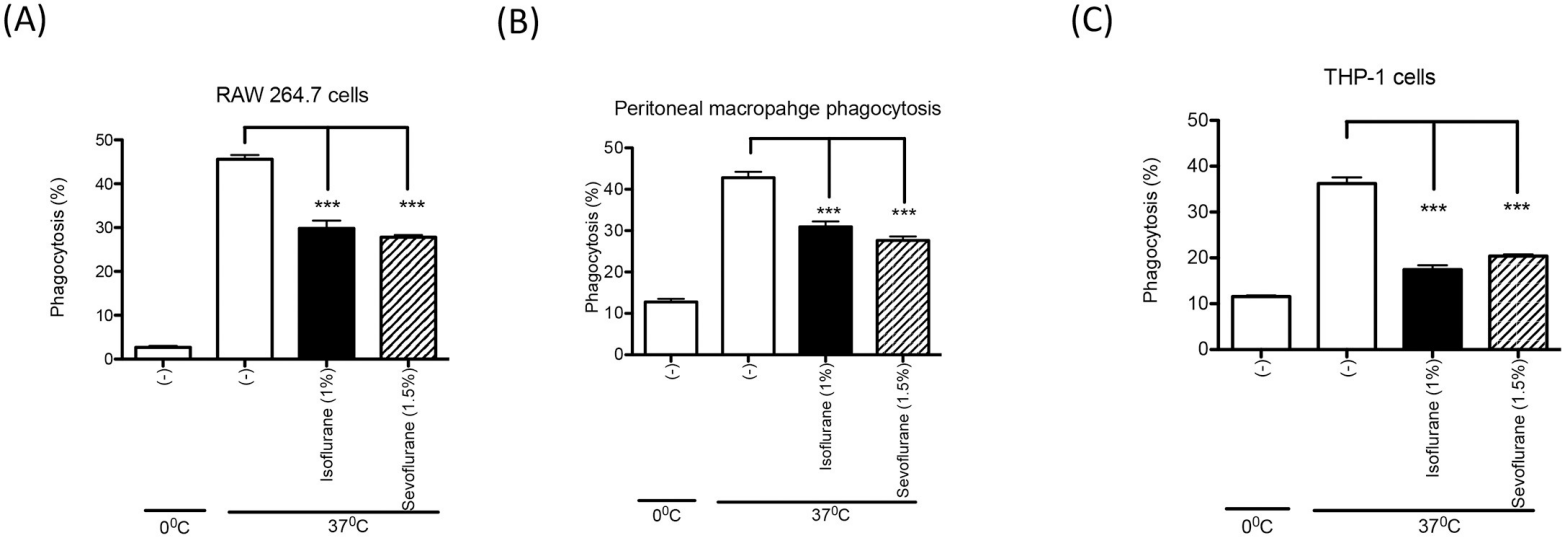
The effect of isoflurane and sevoflurane on *E*. *coli* phagocytosis. RAW264.7 cells (A), thioglycollate-induced mouse peritoneal macrophages (B) and PMA-stimulated THP-1 cells (C) were used to test macrophage phagocytosis of fluorescent *E*. *coli* opsonized with serum under Isoflurane (1%) or Sevoflurane (1.5%). Phagocytosis was assessed using flow cytometry as described in Methods. Data are shown as mean +/- S.D. of quadruplicates. Representative figures from two independent experiments are shown. Statistical analysis was performed using one-way ANOVA with Bonferroni *post hoc* analysis. *** represents p<0.001, respectively.

We tested phagocytosis of IgG opsonized sheep erythrocytes by murine macrophage RAW 264.7 cells in the presence of isoflurane, sevoflurane or propofol. We found that isoflurane (2%) and sevoflurane (3%) attenuated macrophage phagocytosis (Fig 2A). Similarly, isoflurane (1%) and sevoflurane (1.5%) attenuated macrophage phagocytosis (Fig 2A). However, propofol did not inhibit macrophage phagocytosis even at supra-clinical concentration (20 and 100 μM) (Fig 2A). Although the average phagocytosis % at 2% of isoflurane was lower than the one at 1% of isoflurane, there was no statistical significance between the two doses (Fig 2B). Sevoflurane showed a dose-dependent inhibition in phagocytosis (Fig 2B). On RAW 264.7 cells, Mac-1 (CR3) was the major complement receptor, while FcγI and FcγIII were main phagocytic Fc receptors (Fig 2C). FcγΙΙ, though expressed, does not get involved in active phagocytosis on RAW 264.7 cells [12]. The surface expression of Mac-1, FcγI and FcγII/III was not affected by anesthetic exposure (Fig 2C), suggesting that the expression levels of these receptors were not the reason for the reduced phagocytosis by either isoflurane or sevoflurane (Fig 2A). In line with the previous publication [12], blocking Mac-1 attenuated phagocytosis significantly (Fig 2A), confirming that both FcR and Mac-1 work in concert for this phagocytosis mechanism. Using different macrophage cell lines, we also tested the effect of isoflurane and sevoflurane on phagocytosis of serum opsonized *E*. *coli*, which is a clinically relevant phagocytosis assay. Because propofol at both 20 μM and 100 μM did not affect RAW 264.7 cell phagocytosis, we did not test propofol in *E*. *coli* phagocytosis assay. Isoflurane and sevoflurane attenuated macrophage phagocytosis of *E*. *coli* using RAW 264.7 cells, peritoneal macrophages and PMA-stimulated THP-1 cells (Fig 3A–3C).

### Both sevoflurane and isoflurane attenuated Rap1 activation

**Fig 4:**
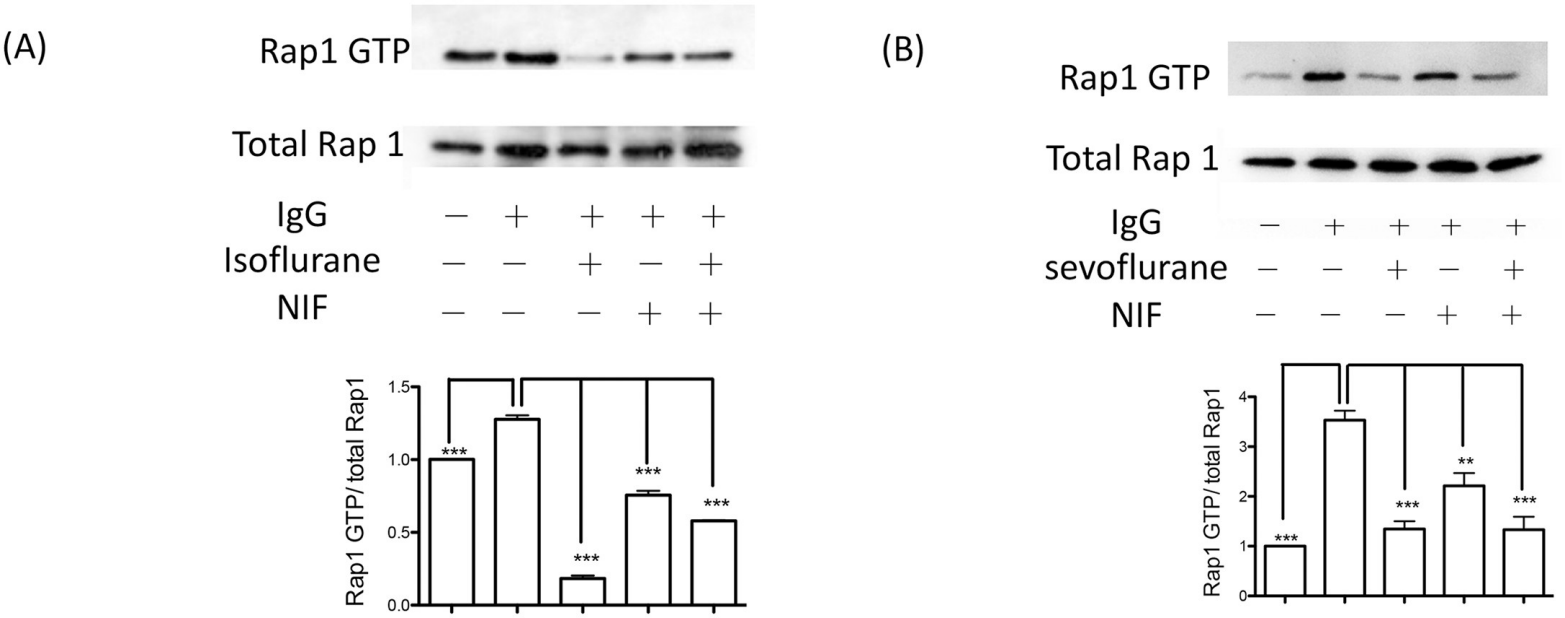
The effect of anesthetics on Rap1 activation of RAW264.7 cells. Rap1 activation was probed using Rap1 activation assay kit as described in the method section. RAW cells were co-incubated either with non-opsonized sheep erythrocytes or IgG opsonized sheep erythrocytes. Rap1 activation was tested with or without (A) isoflurane (1%) and (B) sevoflurane (1.5%). Neutrophil inhibitory factor (NIF) was used at 10 μM. Representative blots are shown from two independent experiments with the same pattern. Immunoreactive proteins were visualized by enhanced chemiluminescence and intensities were quantified with Image J software. Data are shown as mean+/- S.D. of Rap1 GTP/ total Rap1 from three independent measurements. Statistical analysis was performed using one-way ANOVA with Bonferroni *post hoc* analysis. ** and *** represents p< 0.01 and p<0.001, respectively.

Rap1 is a Ras-like small GTPase [19] and a critical molecule involved in IgG- and complement-mediated phagocytosis [20, 21]. The low molecular weight GTPase cycles between an inactive, GDP-bound conformation and an active, GTP-bound conformation. Both isoflurane and sevoflurane exposure attenuated Rap1 activation (Fig 4A–4B). Mac-1 inhibition by neutrophil inhibitor factor (NIF) significantly attenuated Rap1 activation (Fig 4A–4B). We previously demonstrated that isoflurane blocked Mac-1, but sevoflurane did not [22]. Taken together, the inhibition of phagocytosis by isoflurane might be partly due to its inhibitory effect on Mac-1, while sevoflurane likely inhibited macrophage phagocytosis by targeting non-integrin molecules.

### Azi-sevoflurane, not azi-isoflurane showed adduction on Rap1

**Fig 5:**
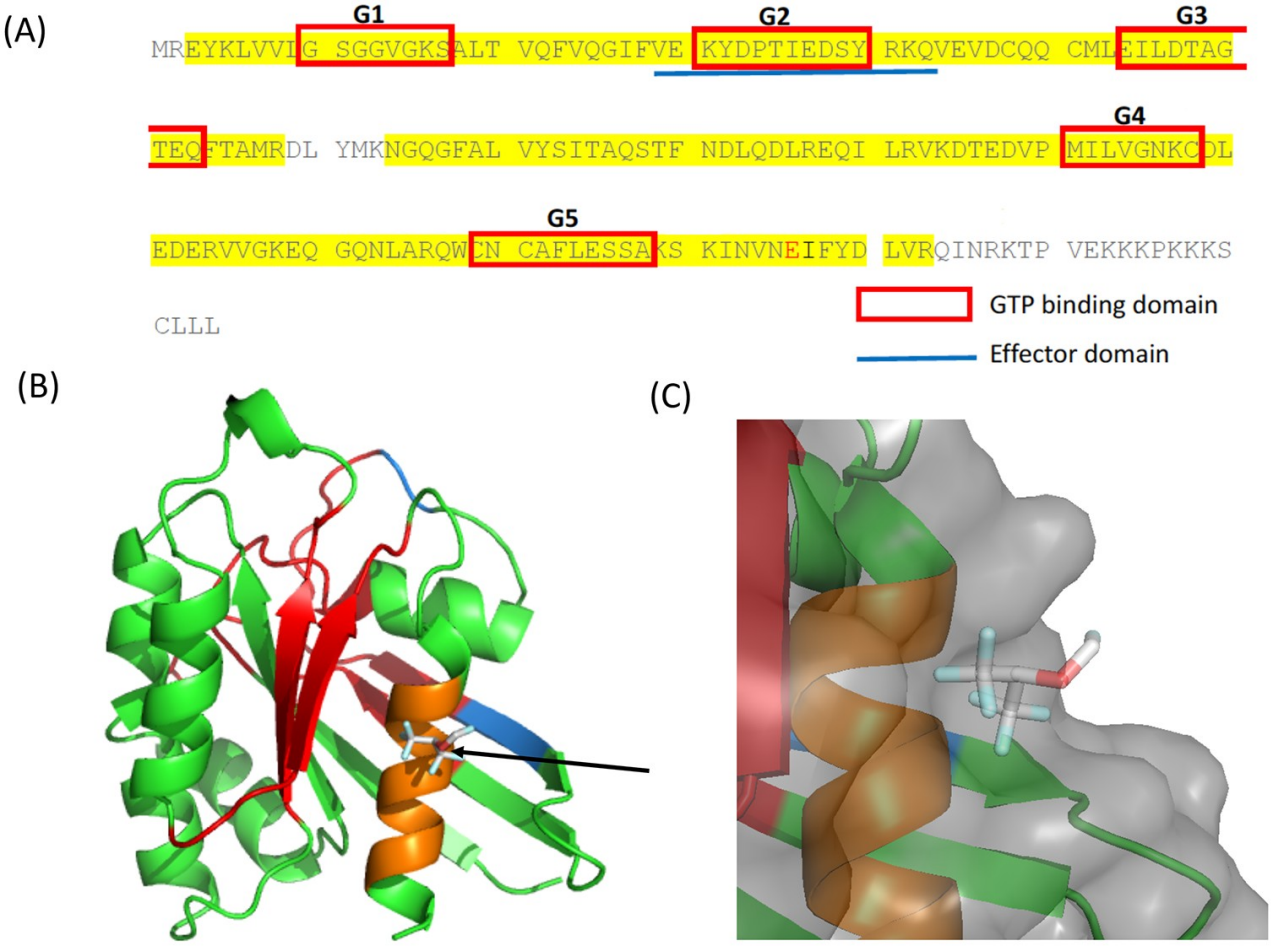
Photolabeling of Rap1 by using azi-sevoflurane. (A) Photolabeling experiment was performed using azi-sevoflurane. Sequences covered by mass spectrometry is highlighted in yellow. The adducted residue is shown in red. Red box denotes high conserved GTP-binding domain among Ras-related proteins. The blue line denotes effector binding site. (B) Rigid docking of sevoflurane was performed on Rap1. Arrow shows docked sevoflurane. GTP binding domains were indicated in red. The effect binding domain was shown in blue (overlapping G2 domain is shown in red). Residues within 4A from the docked sevoflurane were shown in orange. (C) Sevoflurane binding pocket is shown. The surface is shown in light gray.

Because both isoflurane and sevoflurane attenuated Rap1 activation, we hypothesized that they would directly bind to Rap1 and attenuate its activation. Using photoactivatable isoflurane and sevoflurane, we determined whether these anesthetics directly bound to Rap1. While an adducted residue was observed with the use of azi-sevoflurane, there were no adducted residues by azi-isoflurane within the Rap1 covered by sequencing (Fig 5A and S1 Fig). The Rap1 residues 168–178 was not covered by sequencing in azi-isoflurane experiment, and we cannot exclude the possibility that isoflurane binds to this region. Using the adducted residue by azi-sevoflurane as a grid center, we performed rigid docking of sevoflurane on Rap1 and found that sevoflurane fitted at the site, supporting the result of photolabeling experiment (Fig 5B).

### Sevoflurane binding site affected Rap1 activation

**Fig 6:**
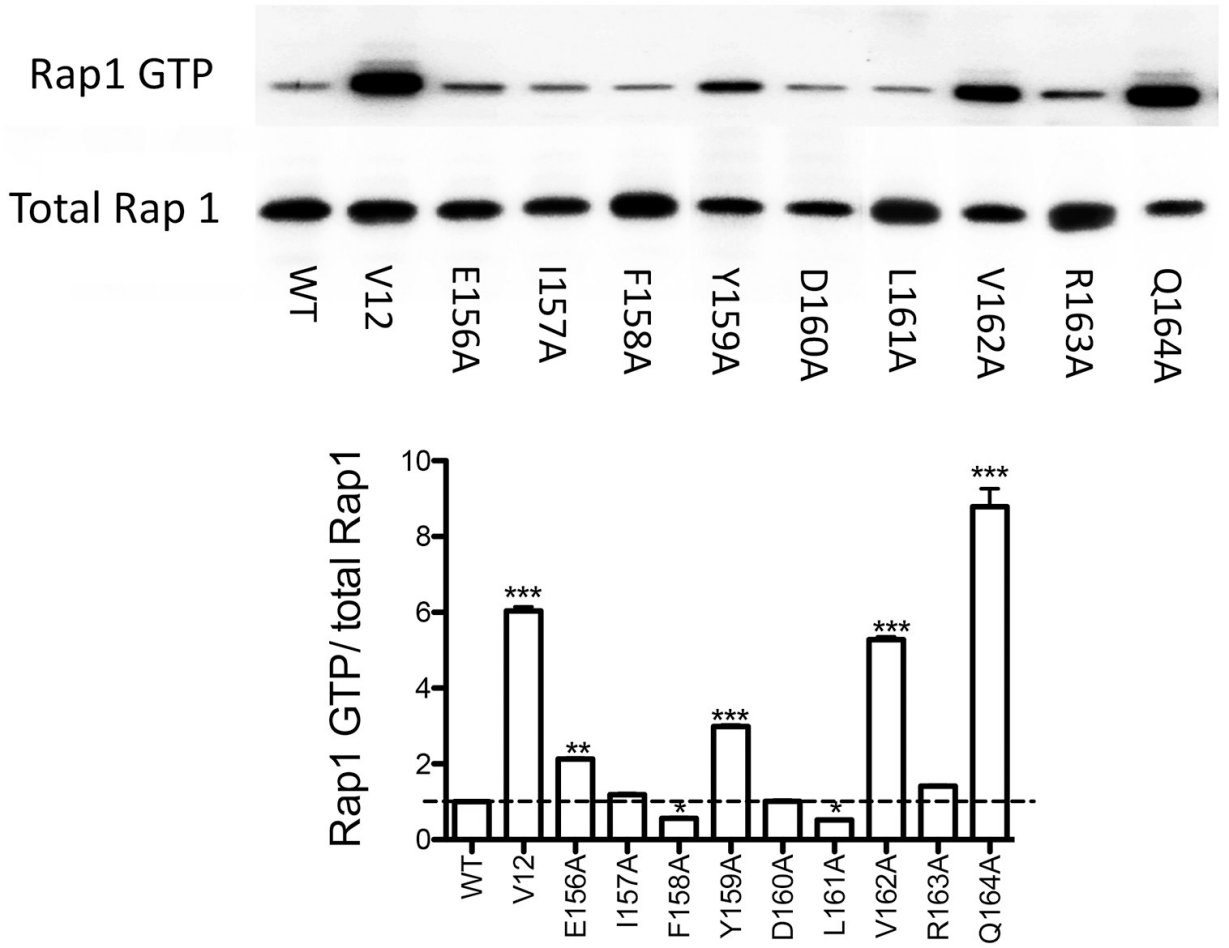
Rap1 activation of alanine mutants at the sevoflurane binding site. Alanine scanning mutagenesis was performed at the sevoflurane binding site using Rap1 pcDNA3.1 plasmid. Mutated plasmid was transiently transfected into HEK293 cells and Rap1 activation was tested. Representative blot is shown from two independent experiments from the same pattern. Immunoreactive proteins were visualized by enhanced chemiluminescence and intensities were quantitated with Image J software. Data are shown as mean+/- S.D. of Rap1 GTP/ total Rap1 from three independent measurements. Statistical analysis was performed using one-way ANOVA with Bonferroni *post hoc* analysis. *, ** and *** represent P<0.05, p< 0.01 and p<0.001, respectively.

To test the functional role of the sevoflurane binding pocket in Rap1, we performed alanine scanning mutagenesis of residues surrounding the docked sevoflurane (S1 Table). Rap1 activation was tested in HEK293T cells transiently transfected with either Rap1 WT or mutants. V12 (G12V) mutant is a known activation mutant [23] and was included as a control. F158A and L161A mutants showed less activation than WT, while E156A, Y159A, V162A and Q164A mutants showed more activation than WT (Fig 6), suggesting that the pocket would serve as an allosteric pocket for Rap1.

### Phagocytic capability of Rap1 mutants

**Fig 7:**
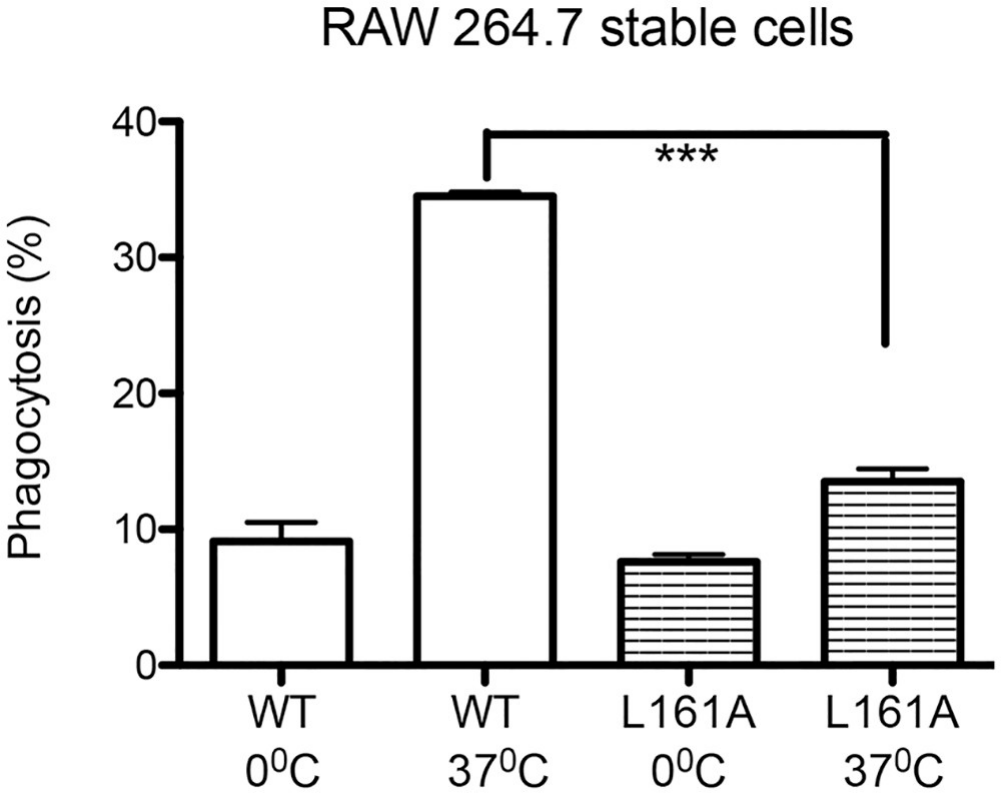
Phagocytosis assay of Rap1 mutant. RAW 264.7 cells were stably transfected with WT and deactivating mutant L161A. Phagocytosis was performed using fluorescence labeled *E*. *coli*. Phagocytosis was assessed using flow cytometry as described in Methods. Data are shown as mean +/- S.D. of quadruplicates. Representative figures from two independent experiments are shown. Statistical analysis was performed using one-way ANOVA with Bonferroni *post hoc* analysis. *** represents p<0.001, respectively.

The data this far suggested that sevoflurane directly attenuated Rap1 activation, and impaired macrophage phagocytosis. To confirm this idea, we made RAW264.7 cells stably transfected with Rap1 WT and L161A mutant. L161A was chosen as an example of deactivating mutant. To mimic clinical scenario of infection, we used serum opsonized *E*. *coli* for phagocytosis assay. Phagocytic activity was significantly impaired in Rap1 L161A mutant than in WT (Fig 7), which verified our idea that sevoflurane interacted with the pocket around L161, and impaired Rap1 activation and macrophage phagocytosis.

## Discussion

In this study we found that volatile anesthetics isoflurane and sevoflurane attenuated macrophage phagocytosis via different mechanisms. The impairment by isoflurane will be explained in part by inhibiting Mac-1 as we previously reported [22]. In addition, isoflurane might interact with molecule(s) in Rap1 signaling cascade. We found that sevoflurane directly bound to small GTPase Rap1 and attenuated its activation. Sevoflurane- Rap1 interaction was at least partly responsible for the phenotype in sevoflurane arm.

We have previously shown that isoflurane attenuated neutrophil function via impairing β2 integrin function and worsened the outcome of experimental polymicrobial abdominal sepsis, but propofol did not [24]. We also reported β2 integrins LFA-1 and Mac-1 as non-canonical isoflurane target [16, 22, 25]. We showed that sevoflurane directly impaired LFA-1, but did not bind to Mac-1 [22]. RAW cell phagocytosis we tested was dependent on Mac-1. In line with this report, our experiment showed that isoflurane, not propofol attenuated macrophage phagocytosis. However, we surprisingly found that sevoflurane also attenuated macrophage phagocytosis. We found that sevoflurane directly bound to and attenuated Rap1, but isoflurane did not. This is the first report of Rap1 as a target of volatile anesthetic. Utilizing photoactivatable anesthetics is a very potent approach to delineate potential anesthetic binding sites. Because crystallization of Rap1 has been reported [18], co-crystallization of sevoflurane with Rap1 will further add to the knowledge of how volatile anesthetics interact with proteins. Because the size and structure of isoflurane (144 cubic angstrom) and sevoflurane (154 cubic angstrom) is similar [26], the difference between isoflurane and sevoflurane in Mac-1 and Rap-1 interaction is not clear, requiring future investigation.

β2 integrins are called “leukocyte integrins” and expressed only on leukocytes. “Inside-out” signal is the intracellular signals triggered by the cell surface receptors to activate integrin-mediated adhesion. Rap-1 activation initiates the activation of these integrins via inside-out signals, and Rap-1 serves as an additional volatile anesthetic target in β2 integrin signaling pathway. Rap1 is expressed on a wide range of cell types and its activation is involved in many biological processes [27], such as platelet adhesion [28]. Previously, we demonstrated that isoflurane and sevoflurane attenuated the activation of platelet receptor integrin αIIbβ3 [29]. The effect of sevoflurane on Rap1 may be partly responsible for this observation. With an increasing appreciation of off-target effects by volatile anesthetics, it is of interest to study the mechanism of how volatile anesthetics modulate various cellular functions via Rap1 in the future, particularly because Rap1 is a widely expressed small GTPase [30].

The inhibition of Rap1 activation impairs phagocyte function and is not ideal in the setting of infection. Information obtained from this study is useful for Rap 1 biology. Several functional residues are known on Rap1. The regions called “switch I” (residues 30–40) and “switch II” (residues 60–76) stabilize the effector-binding site upon GTP binding [31]. All Ras-related proteins contain five highly conserved domains (G1-G5) mediating GTP binding and hydrolysis (Fig 4A). The effector binding domain is also conserved and shown in blue (Fig 4A). However, the binding site of sevoflurane was not on these regions, hinting this to be a newly shown functional site on Rap1. The critical role of C-terminus phosphorylation (S179) in Rap1 activity has been reported [31], but the binding site delineated here does not overlap that area either. Targeting Rap1 at the conserved domains among Ras-related proteins can inhibit Ras-related proteins in general, while targeting specific sites to Rap1 will lead to the development of highly specific Rap1 inhibitors. Allosteric modulators may behave like a dimmer switch rather than acting as on/off switch because allosteric inhibitors cannot be outcompeted compared with orthotopic inhibitors [32]. Thus, this sevoflurane bindings site should be highlighted because it could serve as a potential site for the development of Rap allosteric inhibitors. Rap1 is involved in some of cancer invasion [27, 33]. Furthermore, sevoflurane could be used as a prototype for Rap1 inhibitor.

Surgical procedures have revolutionized medical care and today 45 million inpatient procedures are performed annually in the U.S [34]. About 40 million anesthetics are administered [35]. However, approximately 400,000 SSIs occur each year and are the most common nosocomial infections in surgical patients, occupying approximately 20% of the estimated two million nosocomial infections in the U.S., with the aggregate annual cost of $3.5-$10.1 billion [7]. SSIs can be devastating and are associated with significantly increased morbidities and mortalities. Our findings suggest that volatile anesthetics may not be favorable for macrophage phagocytosis over intravenous anesthetic propofol, the latter of which is a widely used drug in the operating room as well as in the ICU. Although a limited number of clinical studies are available, volatile anesthetic-based general anesthesia was associated with a higher incidence of surgical site infections and postoperative pneumonia than intravenous anesthetic-based general anesthesia in colorectal surgeries and abdominal surgeries, respectively [36, 37]. In the small study in patients undergoing cardiac catheterization, we demonstrated that volatile anesthetics impaired phagocytosis over intravenous anesthetics [38]. More studies in patients undergoing surgery are needed in the future, but it may be important to keep in mind that commonly used anesthetics in surgical procedures may have a negative impact on infection risk. Development of new anesthetics that devoid inhibition of immunological targets (LFA-1, Mac-1, and Rap1) would be the goal for the future.

In conclusion, we found that volatile anesthetics isoflurane and sevoflurane attenuated macrophage phagocytosis in vitro, but intravenous anesthetic propofol did not. We also found that sevoflurane directly attenuated the function of Rap1, a small GTPase critical for phagocytosis. Sevoflurane binding site may serve as a lead pocket of Rap1 allosteric inhibitor.

## Supporting information

S1 Fig Photolabeling of Rap1 by using azi-isoflurane. Rap1 was photolabelled using azi-isoflurane as described in the method section. No adducted residues were noted.
(TIFF)

S1 Table Residues on Rap1 near the docked sevoflurane. (DOCX)

## Abbreviations

CR: complement receptor
DMSO: dimethyl sulfoxide
Escherichia coli: *E*.*coli*
FBS: fetal bovine serum
FcR: Fc receptor
FITC: fluorescein
HRP: horseradish peroxidase
ICU: intensive care unit
IgG: immunoglobulin G
ITPG: isopropylthiol-β-galactoside
LC: liquid chromatography
MS: mass spectrometry
NIF: neutrophil inhibitory factor
PE: phycoerythrin
PFA: paraformaldehyde
PMA: 12-myristate 13-acetate
SSIs: surgical site infections
WT: wild-type
