# Supplementary figures and images for: Volatile anesthetics affect macrophage phagocytosis
Source: PLoS One. 2019 May 9;14(5):e0216163. doi: 10.1371/journal.pone.0216163 (PMC6508649; doi:10.1371/journal.pone.0216163)

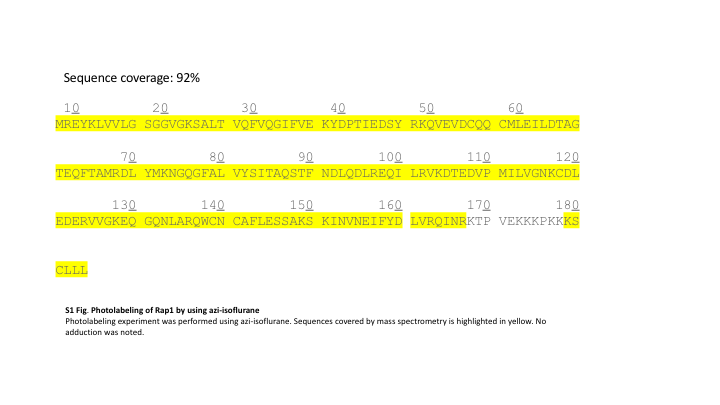

Supplement: S1 Fig — Rap1 was photolabelled using azi-isoflurane as described in the method section. No adducted residues were noted. (TIFF) [file pone.0216163.s001.tiff]
