# Supplementary material for: Volatile anesthetics affect macrophage phagocytosis
Source: PLoS One. 2019 May 9;14(5):e0216163. doi: 10.1371/journal.pone.0216163 (PMC6508649; doi:10.1371/journal.pone.0216163)
Supplement: S1 Table — (DOCX) [file pone.0216163.s002.docx]

**S1 Table. Residues on Rap1 near the docked sevoflurane**

| E156, I157, F158, Y159, D160, L161, V162, R163, Q164, I165 |
| --- |
